# Perioperative tranexamic acid in burn surgery: systematic review and meta-analysis of randomized controlled trials

**DOI:** 10.1016/j.bjane.2025.844719

**Published:** 2025-12-24

**Authors:** Maria Eduarda Molinari, Ramon Huntermann, Julia C. Bernardi, Nicoly Fiorese Andrade, Gustavo Barbosa David, Nicolas Ramos, Caroline de Oliveira Fischer Bacca

**Affiliations:** Centro Universitário para o Desenvolvimento de Alto Vale, Rio do Sul, SC, Brazil

**Keywords:** Antifibrinolytic, Blood loss, Burn patients, Burn surgeries, Tranexamic acid

## Abstract

**Introduction:**

Burn injuries often require surgery, posing challenges due to significant intraoperative blood loss and transfusion risks. Tranexamic Acid (TXA), an antifibrinolytic agent, stabilizes clots and reduces fibrinolysis, with proven efficacy in various surgical settings. However, the benefits in burn patients are yet to be established.

**Methods:**

We performed a systematic review and meta-analysis. PubMed, Embase, and Cochrane databases were searched for Randomized Clinical Trials (RCTs) comparing TXA versus control in burned patients. Risk Ratios (RR) and Mean Differences (MD) with 95% CIs were computed for binary and continuous outcomes, respectively. The primary endpoint of interest was blood loss. Statistical analyses were performed using RStudio software (version 4.2.2). The certainty of the evidence was evaluated using the GRADE approach.

**Results:**

Four RCTs comprising 204 patients were included, with 102 (50%) assigned to the TXA group. The mean patient age across studies ranged from 32.15 to 39.70 years. TXA significantly reduced total blood loss (MD = -183.93 mL; 95% CI: -278.44 to -89.42; p < 0.01), need for packed red blood cell transfusions (RR = 0.42, 95% CI 0.26 to 0.66; p < 0.01), while also improving hematocrit (MD = 3.49%; 95% CI: 1.58 to 5.41; p < 0.01) and hemoglobin levels (MD = 0.87 g.dL^-1^; 95% CI: 0.35 to 1.39; p < 0.01).

**Conclusion:**

In patients with burns, TXA was associated with reduced blood loss and packed cell transfusions. However, certainty is limited by the small number and heterogeneity of available trials

**Registration:**

PROSPERO ID: CRD420251000356. Registered on 07 March 2025.

## Introduction

Burn injuries can cause significant skin damage and often require surgical intervention, which presents a challenge because of substantial perioperative blood loss.^1^ In recent years, early excision and grafting techniques have significantly reduced mortality in cases of severe burns.^2^ However, despite these advancements and the exploration of new treatment approaches, hemorrhage during surgery remains a major concern, resulting in a demand for blood transfusions.^3^^,^^4^ Nevertheless, the effectiveness of blood product transfusion in restoring hemodynamic stability remains associated with increased morbidity and mortality due to both infectious and non-infectious risks.^4^^,^^5^ Currently, intraoperative strategies such as the use of extremity tourniquets, epinephrine, topical thrombin, pre-debridement tumescence with an adrenaline solution, immediate dressing application, and fibrin sealant are available.^6^ However, none of these modalities have consistently demonstrated effectiveness in reducing blood loss. Consequently, in the absence of a clearly superior technique to minimize intraoperative bleeding in patients with severe burns, the administration of blood products remains the only available method to compensate for blood loss.^1^

Tranexamic Acid (TXA) is a lysine analog with antifibrinolytic activity. Its mechanism of action involves the competitive inhibition of plasminogen, a protein responsible for preventing plasmin activation and fibrin degradation.^7^ Its effectiveness has been widely documented in various surgical settings, particularly in procedures related to traumatic injuries.^8^ A meta-analysis evaluating the risk of surgical bleeding across multiple specialties found that TXA significantly reduced perioperative blood loss without increasing the incidence of thromboembolic events.^9^ Several studies have examined the use of TXA in excisional surgeries for patients with severe burns, demonstrating its effectiveness in reducing perioperative blood loss.^10^^,^^11^ Although some meta-analyses have evaluated the intraoperative use of TXA in patients with severe burns, the studies included in these reviews vary in their level of evidence, encompassing a combination of cohort studies and non-Randomized Clinical Trials (RCTs). This study aims to focus exclusively on RCTs, as they provide the highest level of scientific evidence available. In addition to expanding the existing medical literature, our goal is to generate new data to contribute to clinical decision-making.

Therefore, we conducted a systematic review and meta-analysis of RCTs to evaluate whether TXA reduces intraoperative blood loss and transfusion requirements in patients with severe burns. Secondary outcomes included duration of surgical operation and length of hospital stay.

## Methods

This systematic review and meta-analysis was conducted and reported in accordance with the guidelines outlined in the Cochrane Collaboration Handbook for Systematic Reviews of Interventions and the Preferred Reporting Items for Systematic Reviews and Meta-Analysis (PRISMA) Statement.^12^

### Eligibility criteria

Inclusion in this meta-analysis was restricted to studies that met the following eligibility criteria: 1) Randomized controlled trials, 2) Double-blinded design, 3) Comparing TXA versus placebo, 4) Population of burn patients aged over 15 years with at least 20% Total Body Surface Area (TBSA) burned, and 5) Reporting any outcomes of interest. Studies reported as abstracts or conference presentations were excluded.

### Outcome measures

The primary outcomes were blood loss and Packed Red Blood Cell Transfusion (PRBC) requirements. Secondary outcomes included perioperative hemoglobin and hematocrit levels, colloid and crystalloid units, length of hospital stay, and duration of surgical operation. The blood loss was extracted as reported in the original studies, based on each trial’s predefined definitions and measurement methods. Given the variability in how blood loss was assessed across studies, we compiled a summary of the estimation methods employed by each trial in a supplementary table (Supplemental Methods 1).

### Search strategy and data extraction

A comprehensive search was conducted across PubMed, Embase, and Cochrane databases from their inception until February 2025. The following search terms were utilized in all three databases: ("Burn injury" OR "Burn patients" OR "Burn surgery") AND ("Tranexamic acid" OR TXA OR "Antifibrinolytic agent"). The specific search strategy employed for each database can be found in the Supplementary Material.

Additionally, the bibliographies of all included studies and relevant reviews were manually examined for supplementary research. Two researchers (J.C.B and N.F.A) independently evaluated the data using predetermined search criteria and quality assessment techniques. When disagreements arose between these two authors, they were resolved through discussion and consensus, with input from a third author (M.E.M). The protocol for this meta-analysis was registered with PROSPERO on March 7, 2025, and assigned registration number CRD420251000356.

### Quality assessment

The risk of bias in each study was evaluated using the tool recommended by the Cochrane Collaboration Handbook.^12^ We assessed the risk of bias in RCTs using the Risk of Bias in RCTs (RoB-2) tool.^13^ This evaluation was conducted independently by two reviewers (M.E.M and R.H). Additionally, two independent researchers (M.E.M and R.H) assessed the certainty of evidence using the Grading of Recommendations, Assessment, Development, and Evaluations (GRADE) system via the GRADEpro Guideline Development Tool. Any discrepancies were addressed through discussion among the authors after examining the complete articles, leading to a consensus. As per Cochrane recommendations, funnel plots and Egger’s test were not performed due to the number of included studies in each outcome (n < 10).^12^

### Statistical analysis

To compare continuous outcomes between the TXA and control groups, Mean Differences (MD) with 95% Confidence Intervals (95% CI) were calculated. All outcomes were pooled using the inverse variance method under a random-effects model. Risk Ratios (RR) with 95% CI were calculated to compare the incidence of binary outcomes. To address methodological and demographic heterogeneity among the included studies, we implemented a Restricted Maximum Likelihood (REML) random-effects model for all outcomes. We evaluated heterogeneity using three methods: Cochran's *Q* test, I² statistics, and tau-squared. Heterogeneity was reported as low (I² = 0%‒25%), moderate (I² = 26%‒50%), or high (I² > 50%).^14^ Studies with zero events in both arms were excluded from the meta-analysis, in accordance with the Cochrane Handbook.^12^

All statistical analyses were performed using R statistical software (version 4.2.1) using the meta package. Prediction intervals were calculated to account for between-study variability and to provide a range in which the true effect of a future study would be expected to fall. Although not formally included in the GRADE approach, prediction intervals can indirectly inform judgments about imprecision, a key domain in GRADE assessments.

### Sensitivity analyses

A leave-one-out sensitivity analysis was performed for all outcomes with at least I² ≥ 50% and three or more studies to assess the robustness of findings. In this strategy, each study was omitted, and the pooled MD or RR was recalculated for the remaining studies in the analysis.

### Trial sequential analysis

A Trial Sequential Analysis (TSA) was conducted to assess the consistency of the outcomes analyzed, with parameters set at a type 1 error of 5% and a type 2 error of 20%. This analysis was performed using TSA software (Copenhagen Trial Unit, Centre for Clinical Intervention Research, Copenhagen).^15^

## Results

### Study selection and characteristics

**Figure 1:**
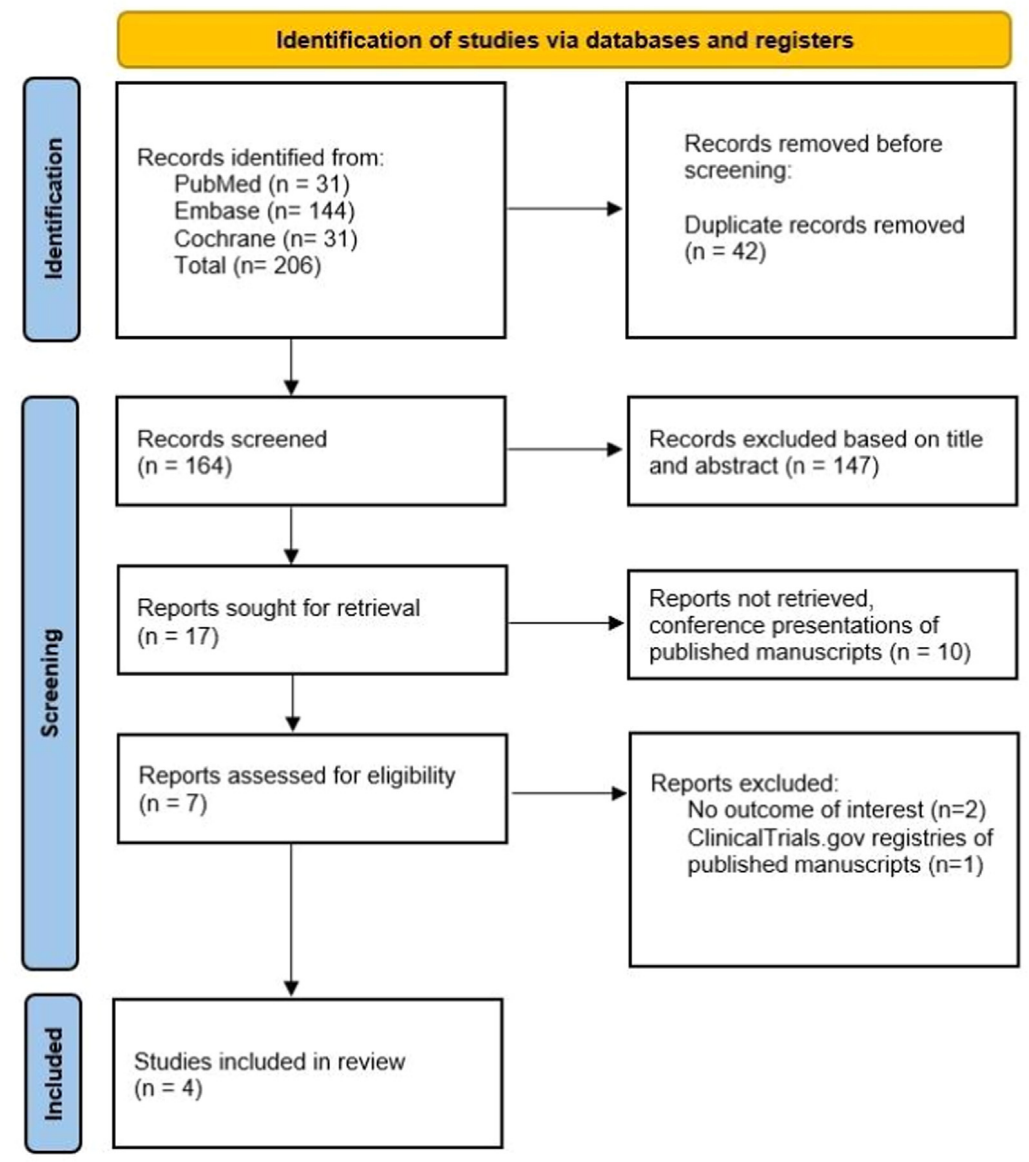
PRISMA flow diagram of study screening and selection.

The initial search in our systematic review produced 206 results. Following the elimination of duplicate entries and screening based on titles and abstracts, 7 full-text articles were evaluated for potential inclusion. Ultimately, 4 RCTs met our inclusion criteria and were incorporated into the analysis. These studies encompassed a combined population of 204 participants, with 102 (50%) assigned to the TXA group. Figure 1 illustrates the details of the study selection process.^16^

The studies encompassed participants with a mean age range of 32.15 to 39.7 years, and 124 (60.78%) of the subjects were male. Ajai et al.’s^17^ research included individuals with third-degree, chemical, and electrical burns affecting less than 30% of their total body surface area upon presentation. The study by Bhatia et al.^18^ focused exclusively on adults with burns covering more than 20% of their total body surface area, excluding those with additional health conditions such as myocardial infarction, unstable angina, or renal/hepatic insufficiency. Cardiel et al.’s^19^ investigation was limited to patients undergoing their first surgical debridement for burn injuries. The research conducted by Naderi et al.^20^ concentrated on patients with severe burns, defined as those affecting over 20% of the total body surface area. A summary of the population characteristics included in this meta-analysis is presented in Table 1.

**Table 1:** Baseline characteristics of included studies.

Study	Design	TXA regimen^a^	Control	Patients (n)	Age^a^ (Years)		Male (n)		Hb transfusion threshold (g.dL^-1^)	BMI^b^ (kg.m^-^²)		Burn-surgery interval (days)	TBSA^b^ (%)		Anesthesia type	Follow-up
					TXA	C	TXA	C		TXA	C		TXA	C		
Ajai 2022	RCT	15 mg.kg^−1^ Preoperatively	10 mL saline IV	30	33.6	30.7	12	14	< 8	21.2	20.9	6	28.4	26.7	General anesthesia	3 months postoperatively
Bhatia 2017	RCT	15 mg.kg^−1^ Preoperatively	25 mL saline IV	50	35.1	36.2	15	14	< 7	20.8	21.6	7	38.6	41.8	General anesthesia	Intraoperative and 24 hours postoperatively
Cardiel 2025	RCT	10 mg.k^g−1^ Preoperatively	10 mL saline IV	30	33.8	35.2	9	11	< 10	27.3	26.2	10	26.5	27.1	NA	Intraoperative and 24 hours postoperatively
Naderi 2025	RCT	10 mg.kg^−1^ bolus + 1 mg.kg^−1^ during surgery per hour.	Saline solution IV	94	39.3	40.1	24	25	Exceeding ABL	24.8	24.5	NA	45.6	43.5	General anesthesia	NA

a Mean.

b Dose of TXA used. ABL, Allowable Blood Loss; BMI, Body Mass Index; C, Control; Hb, Hemoglobin; NA, Not Available; RCT, Randomized Controlled Trial; TBSA, Total Body Surface Area; TXA, Tranexamic Acid.

### Blood loss

**Figure 2:**
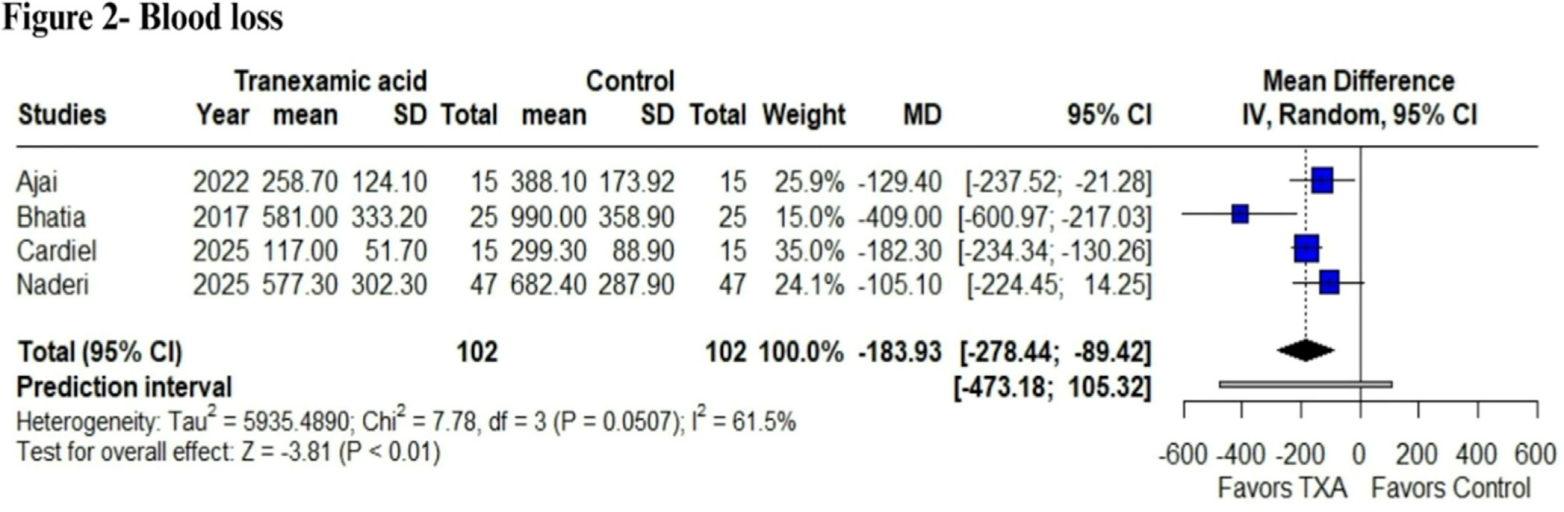
Blood Loss analysis. Comparison of total blood loss between perioperative tranexamic acid administration and control in burn patients undergoing surgical excision. Patients in the TXA group showed a reduced blood loss volume (p < 0.01).

In the pooled analysis, TXA was associated with a significant reduction in blood loss compared with the control (MD = -183.93 mL; 95% CI: -278.44 to -89.42; p < 0.01; I² = 61.5%; Fig. 2). The included studies assessed blood loss at different time points; Bhatia et al.^18^ reported intraoperative blood loss, whereas the other trials measured total perioperative blood loss.

### Laboratory parameters

**Figure 3:**
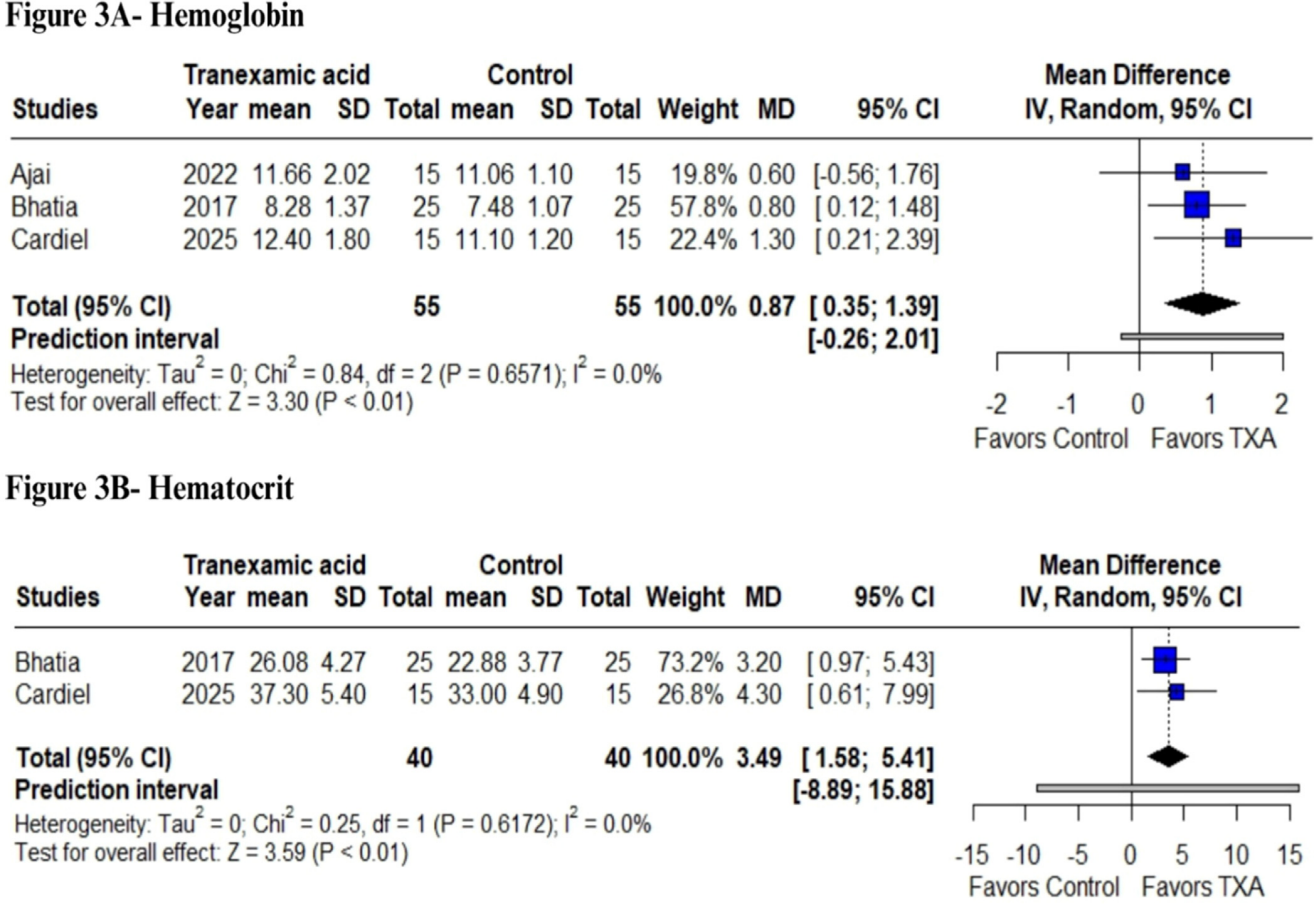
Laboratory parameter analysis. (A) Comparison of hemoglobin (g.dL^−1^) and (B) Hematocrit (%) levels between perioperative tranexamic acid administration and placebo in surgical treatment of burn injuries.

In terms of laboratory parameter outcomes, our analysis revealed a statistically significant increase in hemoglobin concentrations following TXA use (MD = 0.87 g.dL^-1^; 95% CI: 0.35 to 1.39; p < 0.01; I² = 0%; Fig. 3A). As expected, a decrease in hemoglobin levels compared to the baseline was observed after surgery. In the TXA group, variations ranged from -2.06 to -0.69 g.dL^-1^, whereas in the control group, the decline was more pronounced, ranging from -3.02 to -1.24 g.dL^-1^. Similarly, we observed a significant increase in hematocrit levels (MD = 3.49%; 95% CI: 1.58 to 5.41; p < 0.01; I² = 0%; Fig. 3B).

### Fluids

**Figure 4:**
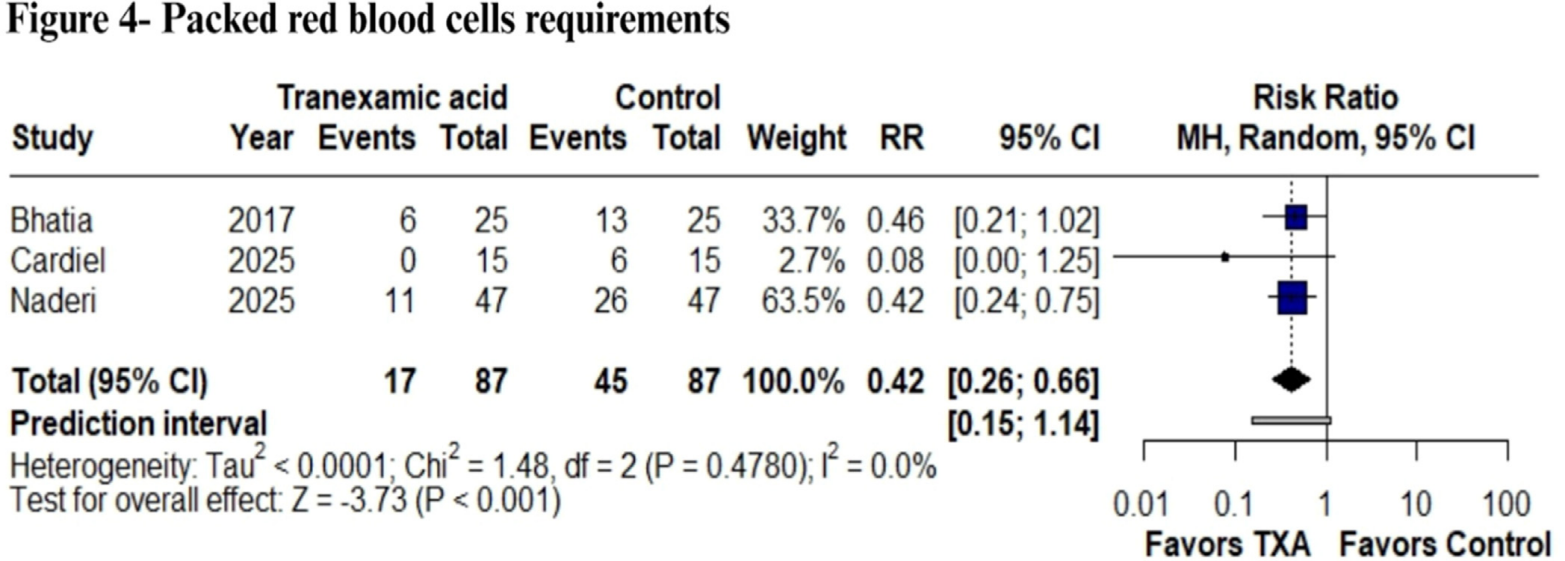
PRBC requirements analysis. Comparison of transfusion requirements between perioperative tranexamic acid administration and placebo in burn surgery. A reduction in transfusion requirements was observed in the TXA group (p < 0.001).

The investigation focused on intraoperative crystalloids and colloids, as well as the need for PRBC transfusions administered during hospitalization. Statistical analyses indicated no significant reduction in the quantity of colloid (MD = -0.25 units; 95% CI: -1.15 to 0.65; p = 0.59; I² = 92.7%) (Fig. S1) or crystalloid (MD = 0.10 units; 95% CI: -0.32 to 0.51; p = 0.64; I² = 58%) (Fig. S2). In contrast, a statistically significant decrease was observed in the number of PRBC requirements. (RR = 0.42; 95% CI: 0.26 to 0.66; p < 0.001; I² = 0%) (Fig. 4).

### Length of hospitalization and duration of surgery

The analysis revealed no statistically significant differences in hospitalization stay (MD = -1.56 days; 95% CI: -4.42 to 1.31; p = 0.29; I² = 82.3%; Fig. S3). Similarly, the reduction in surgical procedure time was not statistically significant (MD = -6.46 min; 95% CI: -15.94 to 3.02; p = 0.18; I² = 47%; Fig. S4).

### Safety outcomes

Regarding safety outcomes, the main concerns with TXA involve Venous Thromboembolic Events (VTE), seizures, and renal complications. However, among the included RCTs, none of the studies reported adverse events related to TXA use, except for postoperative infections described by Naderi et al.,^20^ in which 11 patients in the TXA group and nine in the control group developed infection after the surgical procedure with no statistically significant difference between groups.

### Sensitivity analyses

Sensitivity analyses were performed using a leave-one-out method (Supplemental Table 1). In the analysis of blood loss, the overall effect remained statistically significant in all scenarios, demonstrating the robustness of these findings. Notably, when the study by Bhatia et al.^18^ was omitted, heterogeneity decreased to 0%, suggesting that this study contributed substantially to the observed variability, which may be attributable to differences in the timing of blood loss assessment, as most studies analyzed postoperative bleeding, whereas Bhatia et al.^18^ assessed intraoperative bleeding.

The sensitivity analysis demonstrated that the exclusion of Naderi et al.^20^ reduced heterogeneity to 0% for both hospital stay and surgery duration outcomes. Nonetheless, this exclusion did not change the overall effect estimates, suggesting that although the Naderi et al.^20^ study contributed substantially to statistical heterogeneity, the overall results remained stable and robust (Supplemental Table 1).

### Risk of bias assessment

The Cochrane Collaboration’s tool for assessing RoB-2^13^ was used for quality assessment. No study was considered to have a high risk of bias. Two RCTs were labeled as having some concerns regarding the overall risk of bias.^18^^,^^20^ One trial^20^ demonstrated some concerns regarding missing outcome data due to losses during follow-up. Other concerns were that one trial did not mention any protocol or ethics committee approval^18^ and the other had a retrospective protocol registration.^18^^,^^20^ All other domains were labeled as low risk (Fig. S5). This reflects appropriate randomization, allocation concealment, and blinding of participants, supporting the overall methodological quality of the included trials. The funnel plot analysis was not necessarily due to the number of studies (n < 10)*.*

### Certainty of evidence

According to the GRADE approach, the certainty of the evidence for the evaluated outcomes was initially rated as high, given that all included studies were randomized controlled trials. However, the certainty was subsequently downgraded based on judgments related to risk of bias, inconsistency, imprecision, and potential publication bias. Our GRADE analysis revealed that most outcomes were supported by low or very low certainty of evidence. For blood loss, the evidence indicates a reduction; however, there was some indirectness due to the timing of TXA administration relative to burn injury, which varied across studies and was imprecise with a wide prediction interval. Regarding transfusion requirements, the presence of studies with some concerns of risk of bias and variability in transfusion thresholds contributed to further downgrading. Hemoglobin outcomes were judged as moderate certainty due to imprecision, while hematocrit was rated as low certainty given very serious imprecision. Finally, hospital stay, and duration of surgery were rated as very low certainty, mainly due to high heterogeneity and wide prediction intervals. The GRADE assessment is displayed in Supplemental Table 2.

### Trial sequential analysis

The results were considered conclusive only if the Z-curve crossed the trial sequential monitoring boundaries for benefits or harm. Results that did not cross any of the boundaries indicated that the evidence was insufficient to reach a conclusion, and further studies are warranted. In addition, we documented whether the calculated information size was reached. Outcomes that did not achieve the requisite information size for analysis were noted. A detailed description of TSA analysis is present in the summary of findings table (Table 2). Confirmation was obtained for the results related to reduced blood loss, the need for PRBC requirements, and improved hemoglobin levels. The corresponding images for each TSA analysis are available in the Supplementary Table 3.

**Table 2:** Summary of findings.

**Perioperative Tranexamic Acid in Burn Surgery: Systematic Review and Meta-Analysis of Randomized Controlled Trials**						
**Patients:** Burn patients. **Intervention:** Perioperative intravenous tranexamic acid. **Comparison:** Control						
**Outcomes**	**MD or RR (95% CI)**	**Prediction intervals (95% PI)**	**N° of participants (studies)**	**Certainty of the evidence (GRADE)**	Comments*	**Trial sequential analysis**
Blood loss (mL)	MD = −183.93 (−278.44 to −89.42)	−473.18 to 105.32	204 (4 RCTs)	⨁⨁◯◯ Low^a^^,^^b^^,^^c^	Omitting Bhatia et al. turns I² to 0%. The results were consistent and not dependent on any single study.	Confirmed by TSA; RIS achieved
PRBC requirements	RR = 0.42 (0.26 to 0.66)	0.15 to 1.14	174 (3 RCTs)	⨁⨁◯◯ Low^d^^,^^e^	The heterogeneity was 0%	Confirmed by TSA; RIS achieved
Hemoglobin	MD = 0.87 (0.35 to 1.39)	−0.26 to 2.01	110 (3 RCTs)	⨁⨁⨁◯ Moderate^c^	The heterogeneity was 0%	Confirmed by TSA; RIS achieved
Hematocrit	MD = 3.49 (1.58 to 5.41)	−8.89 to 15.88	80 (2 RCTs)	⨁⨁◯◯ Low^f^	The heterogeneity was 0%	The required information size was exceeded at the first information fraction, preventing TSA boundary rendering.
Hospital stay	MD = −1.56 (0.19 to 0.52)	−0.16 to 0.88	154 (3 RCTs)	⨁◯◯◯ Very low^g^^,^^h^	Omitting Naderi et al. turns I² to 0% without altering the non-significant results.	Not confirmed by TSA; RIS not achieved
Duration of surgery	MD = −6.46 (−15.94 to 3.02)	−39.02 to 26.10	174 (3 RCTs)	⨁◯◯◯ Very low^d^^,^^h^	Omitting Naderi et al. turns I² to 0% without altering the non-significant results.	Not confirmed by TSA; RIS not achieved

The corresponding risk, its 95% Confidence Interval, and its 95% prediction intervals were calculated by R software. CI, Confidence Interval; PI, Prediction Interval; MD, Mean Difference; TSA, Trial Sequential Analysis; RIS, Required Information Size.

a Although heterogeneity was high, all trials favored tranexamic acid. Sensitivity analysis showed that exclusion of Bhatia et al. reduced heterogeneity to 0%, likely due to its intraoperative assessment of blood loss, which overestimated the effect compared with the postoperative evaluations of the other trials.

b The settings in which the intervention was initiated varied considerably, with differences in the timing of TXA administration relative to burn injury and in the dosing regimens across studies. These variations significantly increase the level of indirectness in our assumptions.

c Downgraded once due to the wide prediction interval.

d Downgraded once due to risk of bias, as two of the three studies were rated as having some concerns.

e The transfusion thresholds applied to define the timing of blood transfusion varied considerably across the included studies, which increases variability and contributes to greater inconsistency in the results.

f Downgraded twice due to very serious imprecision.

g Downgraded due to high heterogeneity.

h Downgraded for imprecision due to the fact that the 95% PI around the effect size was large.

⁎ In comments, we describe the results from the leave-one-out analysis.

## Discussion

In this present systematic review and meta-analysis of four RCTs and 204 patients, we evaluated the efficacy of perioperative TXA compared with placebo in patients with burns undergoing surgical treatment. The main findings include: 1) Reduced blood loss, 2) Higher hematocrit levels, 3) Increased hemoglobin levels, and 4) Reduction in the need for PRBC transfusions for burn patients. These findings have important implications for perioperative anesthetic and surgical management in burn patients.

The CRASH trial 2 is a landmark study on the use of TXA in trauma, demonstrating a 9% reduction in the risk of death from acute traumatic hemorrhage when administered within three hours of injury.^21^ However, the CRASH trial excluded patients with burns from its analysis. The rationale for using TXA is its potent antifibrinolytic activity. TXA acts by blocking the activation of plasminogen into plasmin, thereby reducing the breakdown of fibrin clots and helping to stabilize hemostasis.^22^^,^^23^ In addition to its antifibrinolytic effect, some studies suggest it may also have anti-inflammatory properties and support endothelial function, although these mechanisms remain secondary and less well established.^24^

The antifibrinolytic action of TXA appears to provide a pharmacological adjunct to optimize perioperative hemostasis. This is reflected in our primary results, with a statistically significant reduction in blood loss volume in milliliters (MD = -183.93 mL). These findings align with those of a meta-analysis by Fijany et al.,^25^ who also reported reductions in blood loss; however, their analysis included some cohort studies. In contrast, Slob et al.^26^ did not observe a statistically significant reduction in blood loss when analyzing only two RCTs, highlighting how limited sample sizes and methodological heterogeneity can affect pooled estimates. Finally, our TSA confirmed the observed reduction in blood loss as a true positive finding in our meta-analysis, and heterogeneity turned to 0% when excluding Bhatia et al.^18^ likely because it measured intraoperative blood loss, overestimating the effect, whereas the other studies assessed across the perioperative period.

It is crucial to note that blood loss can be quantified using different metrics, such as mL/%TBSA or mL.cm^-2^ of excised area. Ajai et al.^17^ measured blood loss per excised area, and Bhatia et al.^18^ measured blood loss by %TBSA, both showing significant reductions with TXA. Only these two studies used alternative metrics, suggesting that TXA’s benefits may extend beyond total blood loss. Focusing solely on the excised surface area could underestimate overall bleeding, whereas broader measures risk overstating the effect. Therefore, future trials should standardize and combine complementary metrics to provide more meaningful clinical insights.

Additionally, TXA was associated with modest but statistically significant improvements in hemoglobin and hematocrit levels, with mean increases of 0.88 g.dL^-1^ and 3.49%, respectively. These laboratory outcomes reflect better perioperative preservation of red cell mass, which may contribute to improved oxygen-carrying capacity and reduced transfusions. Notably, transfusion requirements were reduced by 58%, underscoring TXA's impact on transfusion thresholds and decision-making during anesthetic management, given that more transfusion is related to higher mortality rates.^27^^,^^28^

The interval between burn injury and surgery, and consequently TXA administration, is pathophysiologically relevant for interpreting our findings. Following a severe burn, patients progress through distinct physiological phases, with an initial period characterized by profound systemic inflammation, increased capillary permeability, and fluid shifts, followed by a proliferative phase with progressive stabilization of the microvasculature.^29^^,^^30^ Early surgery during the hyperpermeability phase may be associated with greater intraoperative blood and fluid loss, potentially amplifying the hemostatic benefits of TXA.^31^^,^^32^ In our analysis, the interval from injury to TXA administration ranged from 6 to ≥ 10 days. Therefore, it is important to be careful when interpreting these findings for urgent repairs and later surgeries. The lack of standardized reporting limits assessment of time-dependent effects, highlighting the need for future trials to stratify outcomes by burn phase or time since injury.

Some arguments have emerged against the use of TXA. Its cost-effectiveness has been questioned in the past, although the literature has extensive findings suggesting it is considered a cost-saving alternative. The underlying reason is related to the side effects and clinical and surgical benefits. Furthermore, TXA can reduce the need for blood transfusions, a costly alternative associated with numerous side effects and complications.^33^^,^^34^ However, despite lowering transfusion requirements, no statistically significant reductions were observed in hospital length of stay, operative time, or crystalloid and colloid fluid use. The absence of effects on other cost-related outcomes implies that financial advantage might primarily result from reducing transfusion requirements rather than decreasing the overall use of perioperative resources.^34^, ^35^, ^36^

Moreover, some authors have raised concerns about increased costs related to the management of potential complications, particularly VTE and seizures.^37^ A limitation of our review is that none of the included trials systematically reported adverse events beyond postoperative infection (reported only by Naderi et al.).^20^ This probably reflects the small sample sizes and short follow-up periods of the available RCTs, which restrict the ability to detect and quantify such complications. Importantly, the lack of reported events should not be equated with safety evidence, given the pharmacological profile of TXA and the hypercoagulable state in patients with burns.^38^^,^^39^ Future trials must consistently monitor and report VTE and other clinically relevant adverse events, especially with longer follow-up periods.

Regarding clinical implications, our findings suggest that the use of TXA in burn surgery may help reduce packed cell transfusions with a reduction in blood loss, contributing to more efficient perioperative management. It would be valuable to explore, through future meta-regression analyses, whether factors such as patient age, sex, dose, and extent of TBSA involvement modify the effect of TXA on blood loss. A higher TXA dose or larger burn areas might be associated with greater benefit, but these relationships can only be reliably assessed once a sufficient number of studies (> 10) are available. Thus, its use should be individualized, and further high-quality studies are needed to confirm its efficacy and safety in this population.

This meta-analysis has several limitations that should be acknowledged. First, the small number of included trials and their limited sample sizes reduced the overall robustness, resulting in low statistical power and limiting the strength and generalizability of our findings. Second, substantial heterogeneity in patient characteristics, burn severity, surgical techniques, TXA dosing regimens, and differing transfusion thresholds across studies may have contributed to the variability in the observed effects. Third, the absence of individual patient-level data prevented more granular subgroup evaluations of interest and the exploration of potential effect modifiers through meta-regression analyses. Fourth, the limited number of studies did not allow us to formally assess publication bias using funnel plots or Egger tests. Finally, safety outcomes such as venous thromboembolism and seizures, in addition to other clinically meaningful outcomes such as mortality, were not systematically reported by the included RCTs, thereby limiting our ability to evaluate the safety profile of TXA in patients with burns.

## Conclusion

In available RCTs, TXA was associated with reduced intraoperative blood loss, improved hemoglobin and hematocrit levels, and fewer PRBC transfusions in burn surgery. Certainty is limited; further high-quality, adequately powered studies with standardized dosing and comprehensive safety assessments are needed.

## Authors’ declaration

*AI assistance disclosure:* No artificial intelligence tools, software, or models were used in the generation, writing, analysis, or review of this manuscript.

## Data availability statement

The data associated with the paper are available in PubMed, Cochrane and Embase.

This research did not receive any specific grant from funding agencies in the public, commercial, or not-for-profit sectors.

The datasets generated and/or analyzed during the current study are available from the corresponding author upon reasonable request.

## Funding

This research did not receive any specific grant from funding agencies in the public, commercial, or not-for-profit sectors.

## Authors’ contributions

The authors confirm contribution to the paper as follows: Conceptualization: Maria Eduarda Molinari; Formal analysis: Ramon Huntermann, Maria Eduarda Molinari, Julia Cremonini Bernardi, Caroline de Oliveira Fischer Bacca; Methodology: Ramon Huntermann, Nicoly Fiorese Andrade, Maria Eduarda Molinari; Writing – original draft: Gustavo David Barbosa, Nicolas Ramos, Ramon Huntermann, Maria Eduarda Molinari and Caroline de Oliveira Fischer Bacca.

All authors read and approved the final version of the manuscript.

## Conflicts of interest

The authors certify that there is no conflict of interest with any financial organization regarding the material discussed in the manuscript.

## Appendix Supplementary materials

Image, application 1

## Footnotes

Supplementary material associated with this article can be found in the online version at doi:10.1016/j.bjane.2025.844719.
