# Supplementary material for: Perioperative tranexamic acid in burn surgery: systematic review and meta-analysis of randomized controlled trials
Source: Braz J Anesthesiol. 2025 Dec 24;76(2):844719. doi: 10.1016/j.bjane.2025.844719 (PMC12830265; doi:10.1016/j.bjane.2025.844719)
Supplement: Supplementary file 1 [file mmc1.docx]

**SUPPLEMENTARY MATERIAL**

**Table of Contents**

**Supplemental Methods 1** – Detailed how blood was calculated in each study.

**Supplemental Methods 2** – Search strategy.

**Supplemental Figure S1** – Colloid units were not significantly different between groups (p = 0.59).

**Supplemental Figure S2** – Crystalloids units were not significantly different between groups (p = 0.64).

**Supplemental Figure S3** – Hospital stays not significantly different between groups (p = 0.29).

**Supplemental Figure S4** – Duration time of surgical operation was not significantly different between groups (p = 0.18).

**Supplemental Figure S5 –** Quality assessment with the Cochrane tool for assessing risk of bias in randomized trials (RoB 2).

**Supplemental Table 1** – Leave one out analysis for all outcomes. (A- Blood loss B- Length of hospitalization(days), D- duration of surgery(min).

**Supplemental Table 2** – Grading of Recommendations, Assessment, Development and Evaluations (GRADE).

**Supplemental Table 3** – Trial sequential analysis.

**Supplemental link-** Google sheets which contain the complete extraction of data from each included study.

**Supplemental Methods 1** – Detailed how blood was estimated in each study.

| **Study** | **Blood Loss estimation** |
| --- | --- |
| Ajai et al. | Using the formula described by Gross |
| Bhatia et al. | Using the formula described by Gross |
| Cardiel et al. | Volume of blood aspirated and the weight of the gauze used. |
| Naderi et al. | Using the formula described Herndon |

**Supplemental Methods 2** – **Search strategy**

Search strategy applied in PubMed, EMBASE, and Cochrane Library:

PubMed:

("Burn injury" OR "Burn patients" OR "Burn surgery" OR "Thermal injury" OR "Burn wound" OR "Severe burns" OR "Major burns" OR "Burn trauma" OR "Burned patients") AND ("Tranexamic acid" OR TXA OR "Antifibrinolytic agent" OR "trans-4-(Aminomethyl)cyclohexanecarboxylic acid" OR "Cyclokapron" OR "Hemostatic agent")

EMBASE:

('burn injury' OR 'burn patients' OR 'burn surgery' OR 'thermal injury' OR 'burn wound' OR 'severe burns' OR 'major burns' OR 'burn trauma' OR 'burned patients') AND ('tranexamic acid' OR txa OR 'antifibrinolytic agent' OR 'trans-4-(aminomethyl)cyclohexanecarboxylic acid' OR 'cyclokapron' OR 'hemostatic agent')

Cochrane library:

("Burn injury" OR "Burn patients" OR "Burn surgery" OR "Thermal injury" OR "Burn wound" OR "Severe burns" OR "Major burns" OR "Burn trauma" OR "Burned patients") AND ("Tranexamic acid" OR TXA OR "Antifibrinolytic agent" OR "trans-4-(Aminomethyl)cyclohexanecarboxylic acid" OR "Cyclokapron" OR "Hemostatic agent")

**Supplemental Figure S1 –** Colloid units were not significantly different between groups (p = 0.59).

**
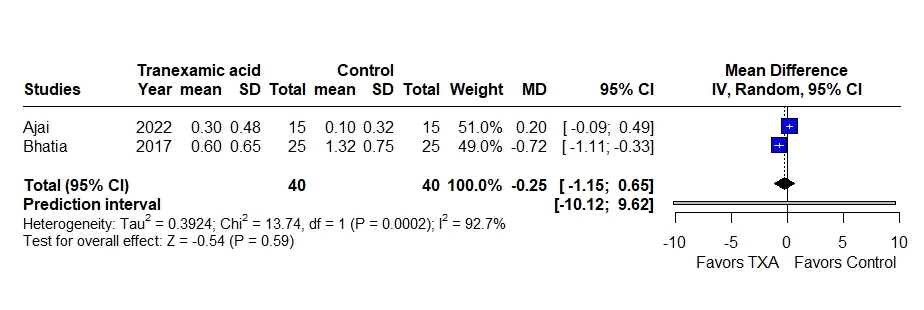
Supplemental Figure S2 –** Crystalloids units were not significantly different between groups (p = 0.64).

**
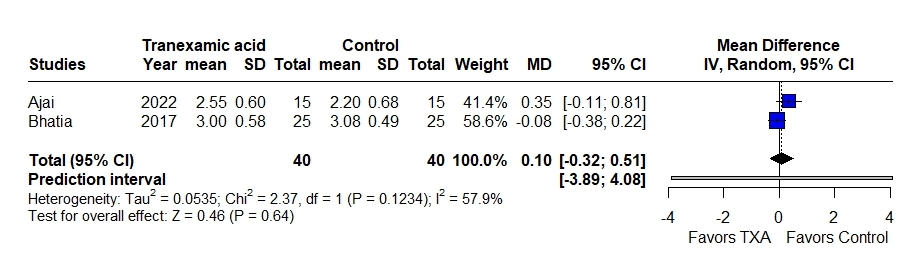
Supplemental Figure S3** – Hospital stay not significantly different between groups (p = 0.29).

**
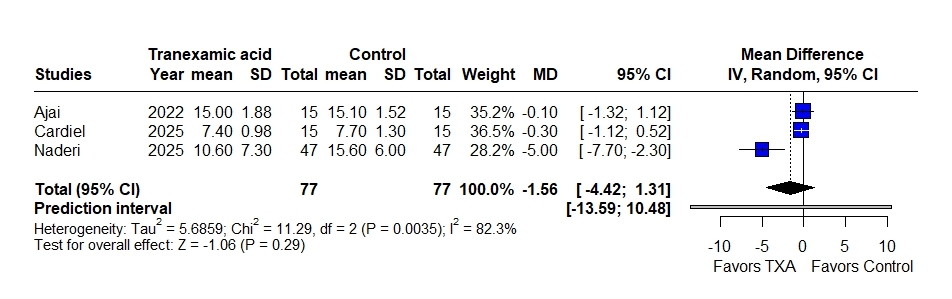
Supplemental Figure S4** – Duration time of surgical operation was not significantly different between groups (p = 0.18).

**
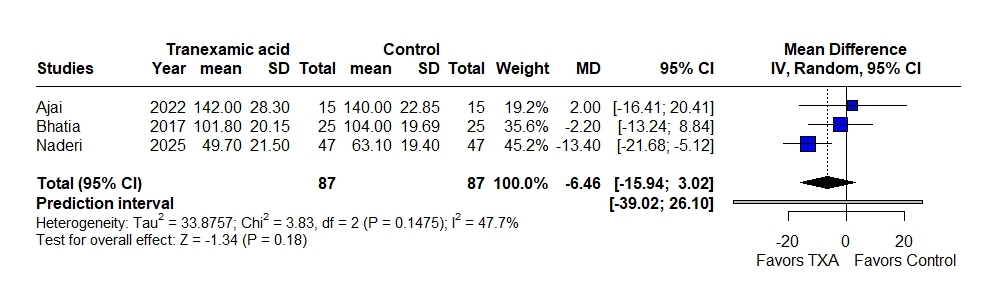
**

**Supplemental Table 1** – Leave one out analysis for all outcomes.

1. **Blood loss, leave-one out**


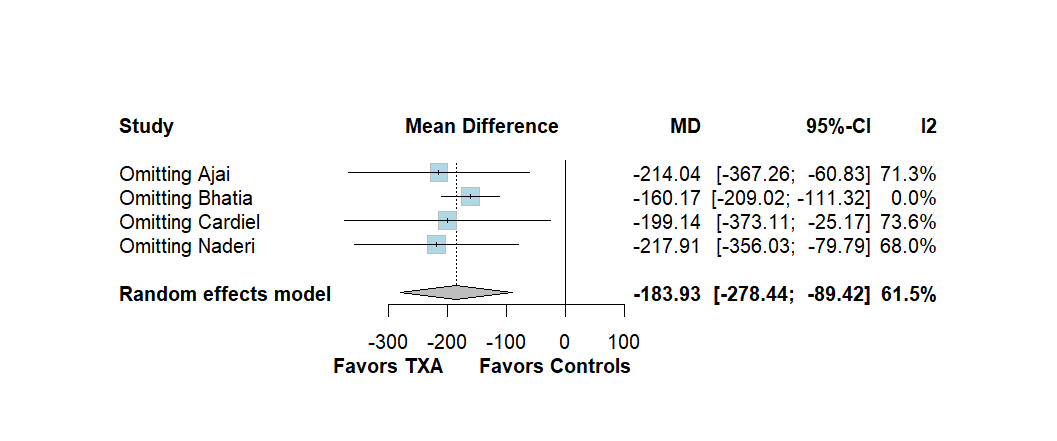


1. **Hospital stay (days), leave-one out**

**
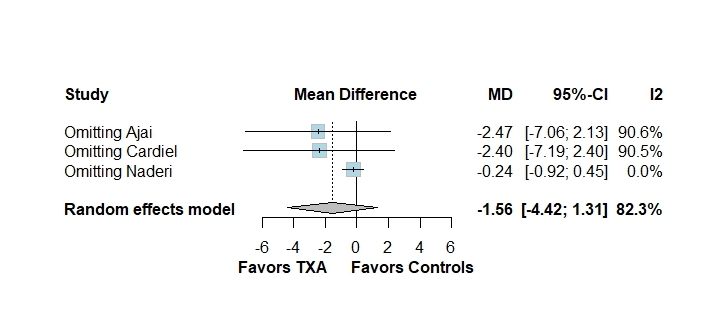
**

1. **Duration of surgery (min), leave-one out**


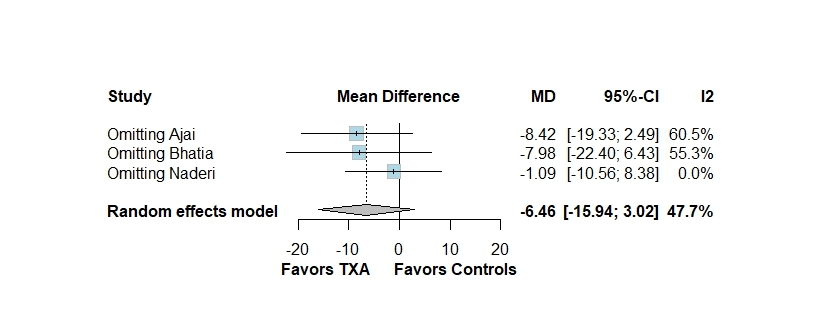


**Supplemental Figure S5 –** Quality assessment with the Cochrane tool for assessing risk of bias in randomized trials (RoB 2).


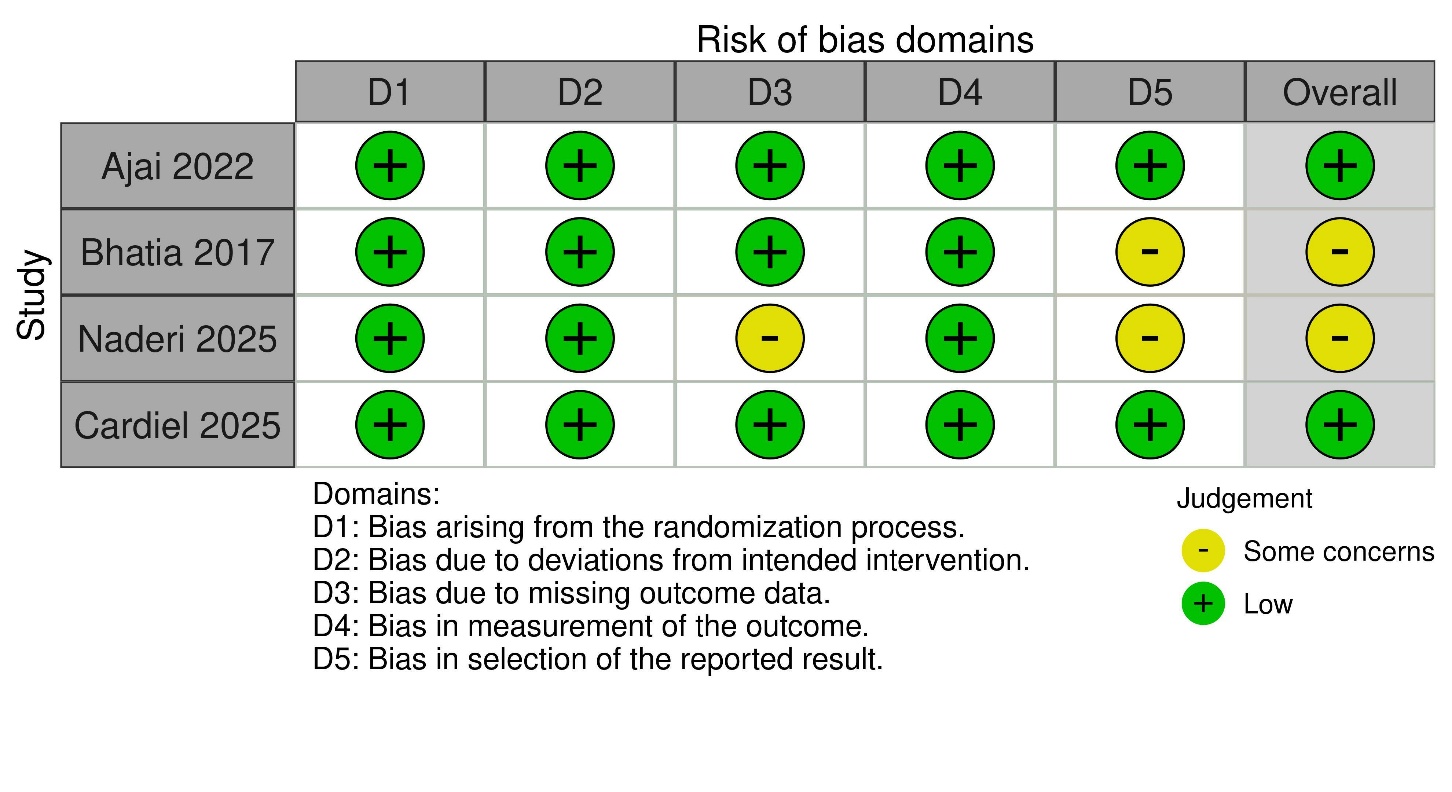


**Supplemental Table 2 –** Grading of Recommendations, Assessment, Development and Evaluations (GRADE).

| **Outcomes** | **№ of participants (studies)** | **Certainty of the evidence (GRADE)** | **Relative effect (95% CI)** | **Anticipated absolute effects** | |
| --- | --- | --- | --- | --- | --- |
|  |  |  |  | **Risk with Control** | **Risk difference with Tranexamic Acid** |
| Blood loss assessed with: mL | 204 (4 RCTs) | ⨁⨁◯◯ Low^a,b,c^ | - | The mean blood loss was **658.17** mL | MD **183.93 mL lower** (278.44 lower to 89.42 lower) |
| PRBC requirements assessed with: n | 174 (3 RCTs) | ⨁⨁◯◯ Low^d,e^ | **RR 0.42** (0.26 to 0.66) | 517 per 1.000 | **300 fewer per 1.000** (383 fewer to 176 fewer) |
| Hemoglobin assessed with: g/dL | 110 (3 RCTs) | ⨁⨁⨁◯ Moderate^c^ | - | The mean Hemoglobin was **9.41** g/dL | MD **0.87 g/dL higher** (0.35 higher to 1.39 higher) |
| Hematocrit assessed with: % | 80 (2 RCTs) | ⨁⨁◯◯ Low^f^ | - | The mean Hematocrit was **26.68** % | MD **3.49 % higher** (1.58 higher to 5.41 higher) |
| Hospital stay assessed with: Days | 154 (3 RCTs) | ⨁◯◯◯ Very low^g,h^ | - | The mean Hospital stay was **13.32** days | MD **1.56 days lower** (4.42 lower to 1.31 higher) |
| Duration of surgery assessed with: Min | 174 (3 RCTs) | ⨁◯◯◯ Very low^d,h^ | - | The mean duration of surgery was **88.11** min | MD **6.46 min lower** (15.94 lower to 3.02 higher) |
| **CI:** confidence interval; **MD:** mean difference; **RR:** risk ratio | | | | | |

Explanations:

a. Although heterogeneity was high, all trials favored tranexamic acid. Sensitivity analysis showed that exclusion of Bhatia et al. reduced heterogeneity to 0%, likely due to its intraoperative assessment of blood loss, which overestimated the effect compared with the postoperative evaluations of the other trials

b. The settings in which the intervention was initiated varied considerably, with differences in the timing of TXA administration relative to burn injury and in the dosing regimens across studies. These variations significantly increase the level of indirectness in our assumptions.

c. Downgraded once due to the wide prediction interval

d. Downgraded once due to risk of bias, as two of the three studies were rated as having some concerns.

e. The transfusion thresholds applied to define the timing of blood transfusion varied considerably across the included studies, which increases variability and contributes to greater inconsistency in the results

f. Downgraded twice due to the very serious imprecision

g. Downgraded due to high heterogeneity

h. Downgraded for imprecision due to the fact that the 95% PI around the effect size was large.

**Supplemental Table 3** – Trial sequential analysis
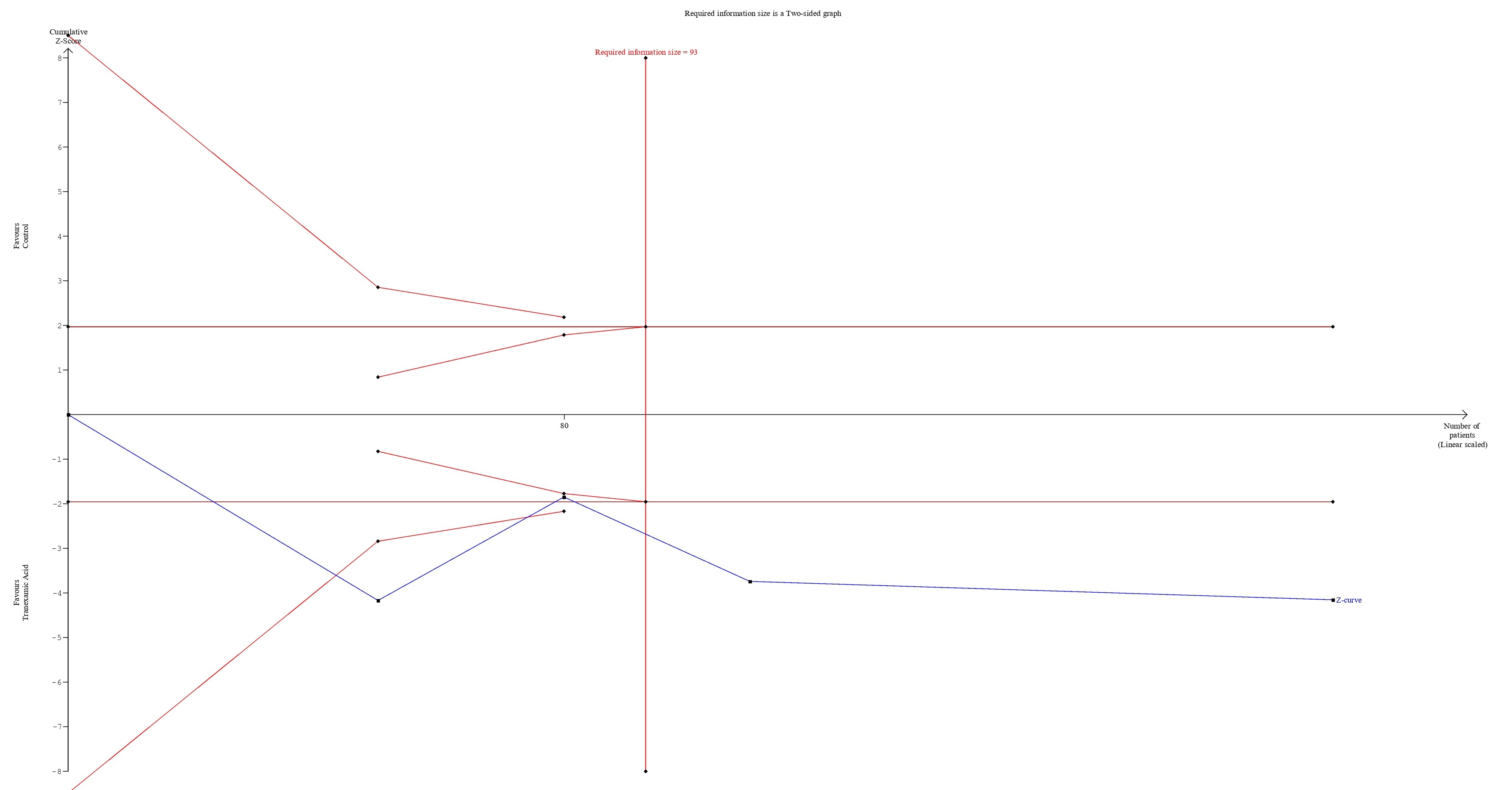
:

Supplementary Figure S6: Blood loss.


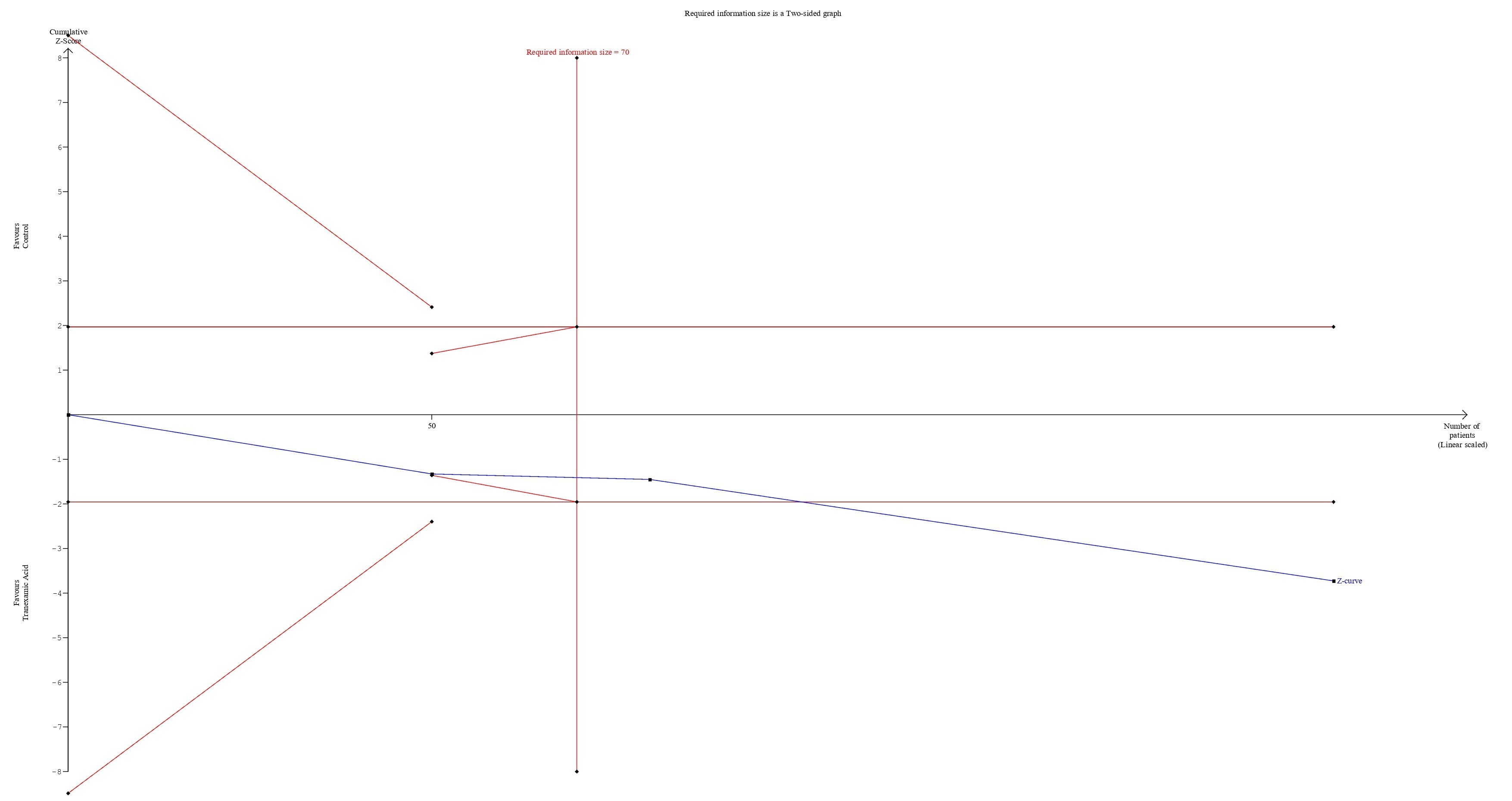
Supplementary Figure S7: Packed red blood cells requirements.

Supplementary Figure S8: Hemoglobin.
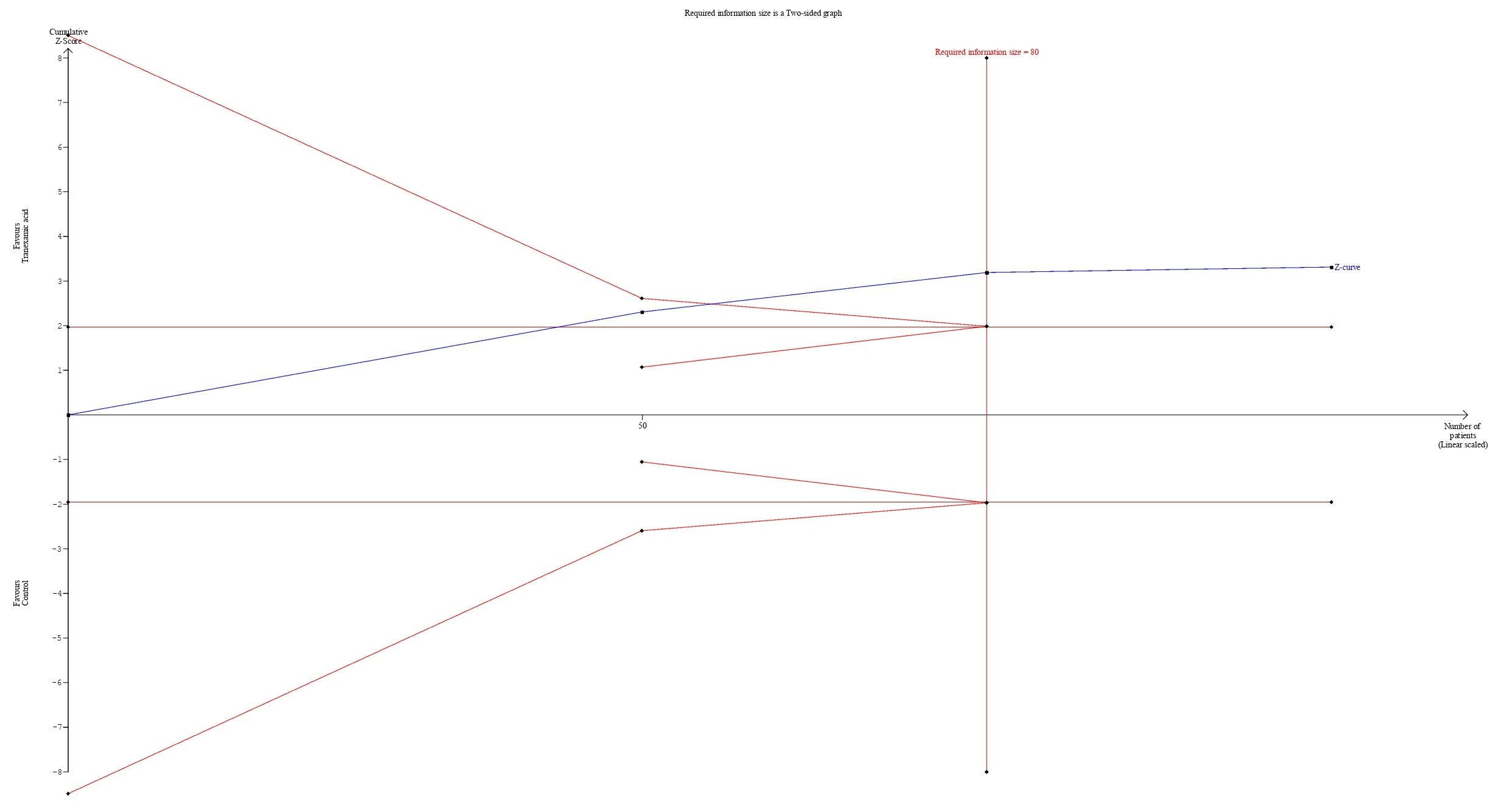


Supplementary Figure S9: hematocrit.


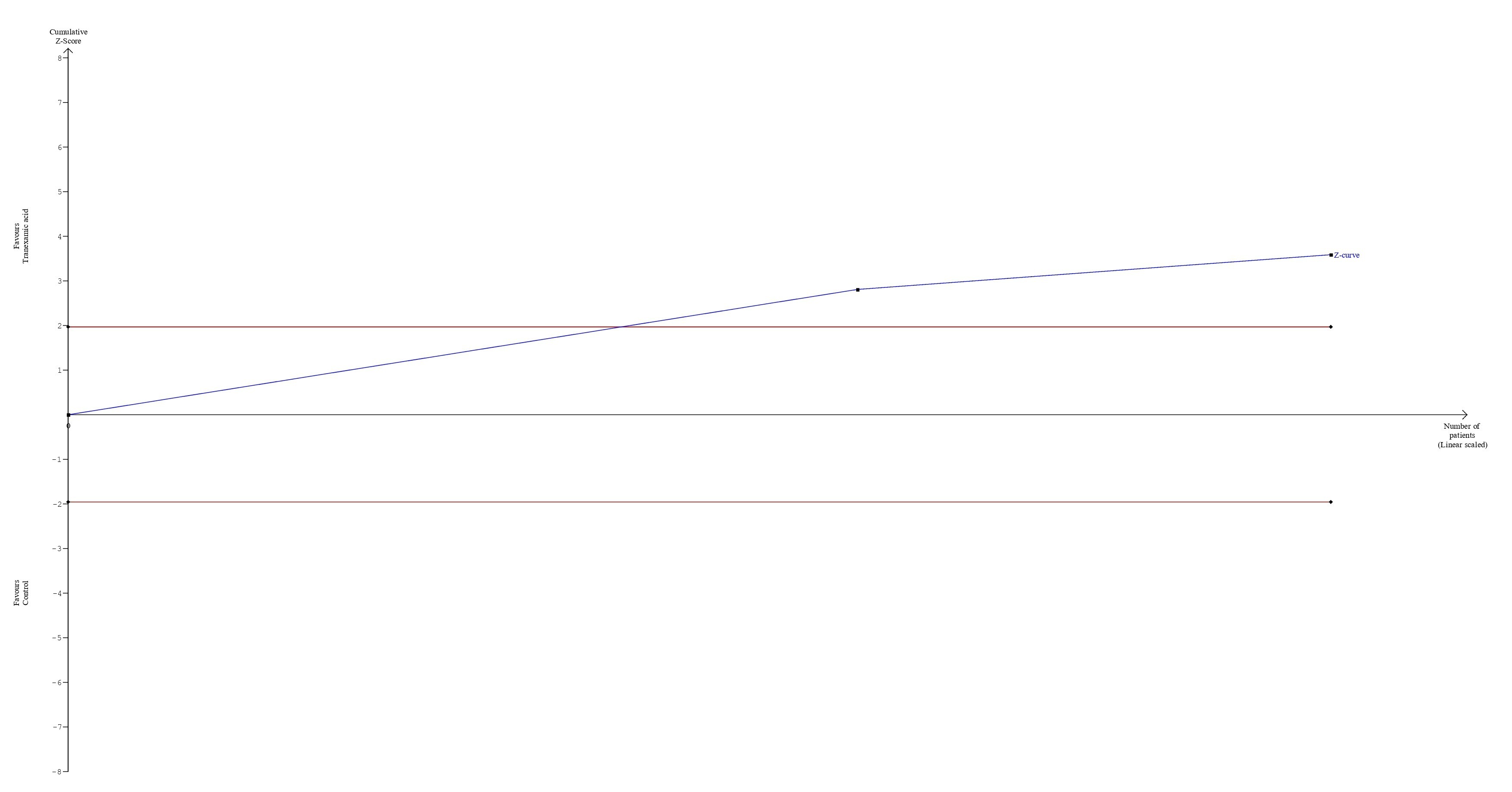
The required information size was exceeded at the first information fraction, preventing TSA boundary rendering.


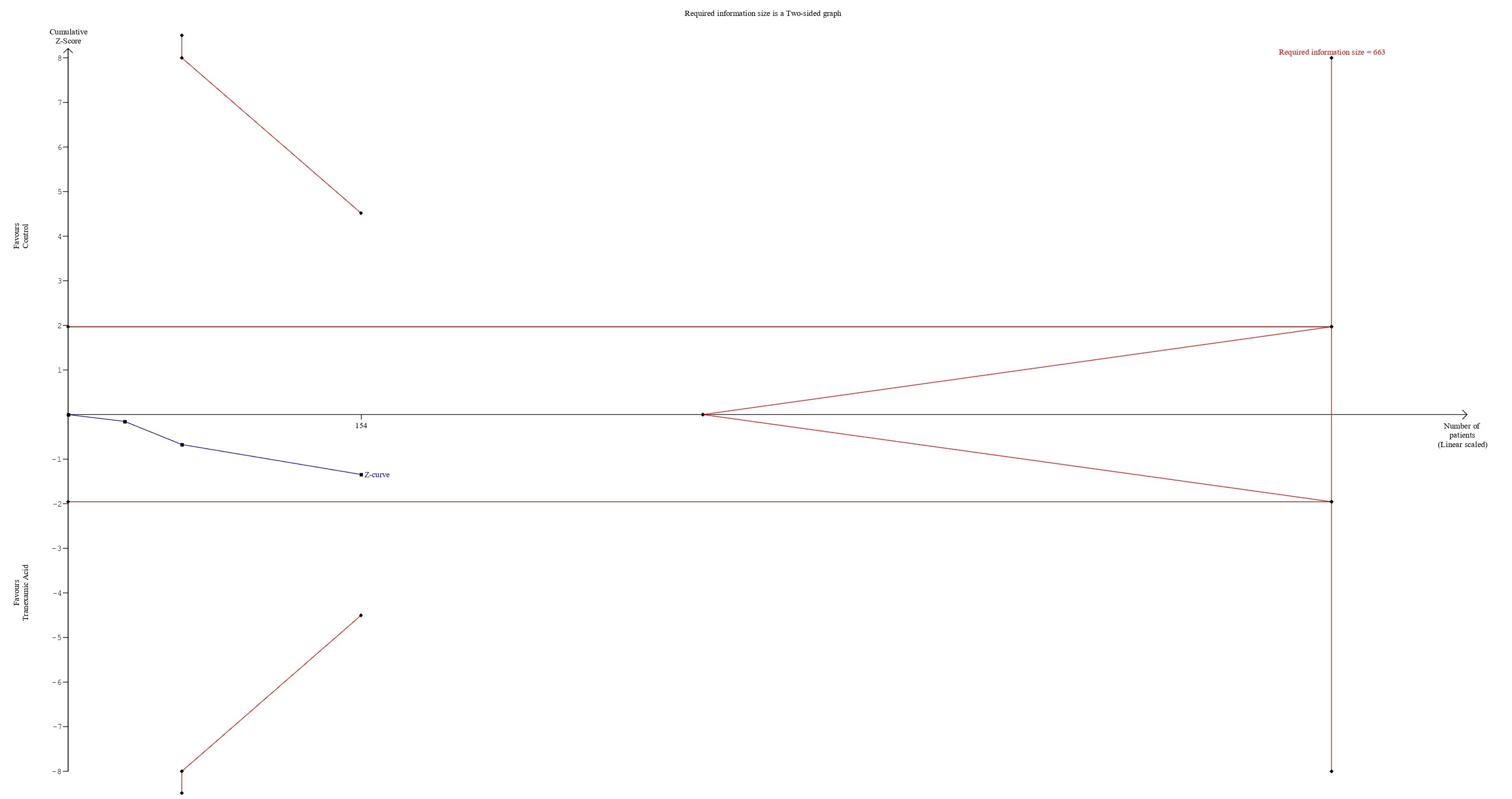
Supplementary Figure S10: Lenght of hospital stay.


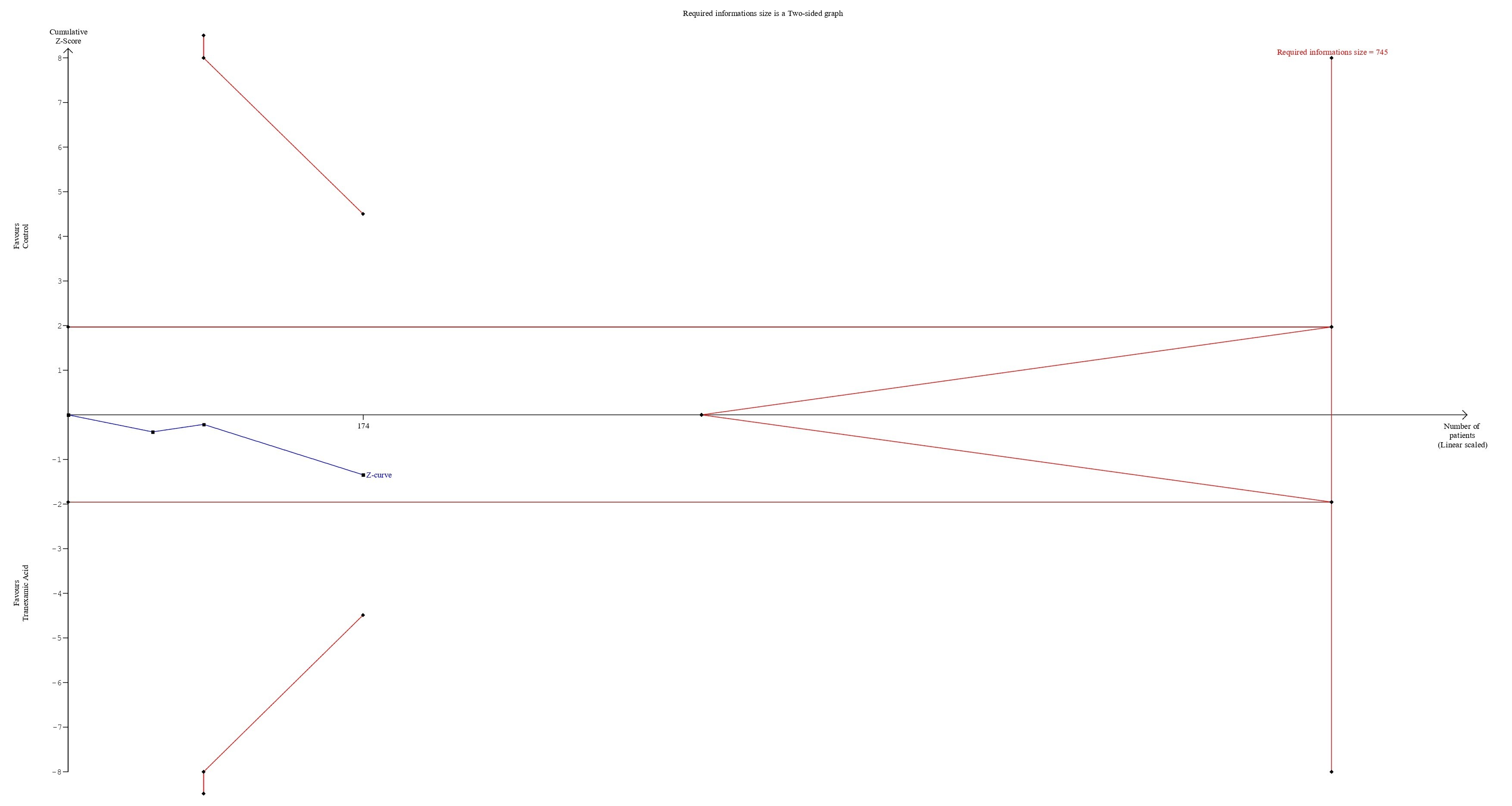
Supplementary Figure S11: Duration of surgery.

Supplementary: Colloids

Did not reach the required percentage of the information size necessary for analysis.

Supplementary: Crystalloids

Did not reach the required percentage of the information size necessary for analysis.

**Supplemental link** – Google sheets which contain the complete extraction of data from each included study.

<https://docs.google.com/spreadsheets/d/1Qy7BC7NW7fu3vIuHEtx8HhyJ2fGG4_Ql/edit?usp=sharing&ouid=117579634310324607999&rtpof=true&sd=true>
